# The Efficiency of the BTR-Pen System in Removing Different Types of Broken Instruments from Root Canals and Its Effect on the Fracture Resistance of Roots

**DOI:** 10.3390/ma15175816

**Published:** 2022-08-24

**Authors:** Merve Dulundu, Dilek Helvacioglu-Yigit

**Affiliations:** 1 Faculty of Dentistry, Kocaeli University, Kocaeli 41190, Turkey; 2 College of Dental Medicine, Qatar University, Doha P.O. Box 2713, Qatar

**Keywords:** broken instrument removal, BTR-Pen, K-file, NiTi rotary instrument, ultrasonic

## Abstract

The study aimed to evaluate the efficiency of the BTR-Pen system in removing different types of instrument fragments from root canals and to assess its effect on fracture resistance of the roots after the removal of the instruments. One hundred thirty human teeth were divided into 10 groups (2 control groups and 8 study groups) according to the localization and type of the fractured fragment as well as the retrieval techniques. Broken instruments were extracted either with BTR-Pen system loops or removed using solely ultrasonic tips. The success rate of instrument removal and consumed time were recorded. All the teeth were subjected to a load at a 1 mm/min rate in a universal testing machine for mechanical testing. The success of removing broken instruments using the BTR-Pen and ultrasonic was 86.7% and 83.3%, respectively (*p* > 0.05). When the time is compared, the BTR-Pen system (23.97 ± 8.35 min) showed similar results to that of the ultrasonic technique (24.1 ± 8.28 min) (*p* > 0.05). The BTR-Pen group required less force to fracture than the ultrasonic group (*p* = 0.024). In conclusion, the BTR-Pen and ultrasonic groups showed no significant difference in terms of the success rate and removal time. The roots that underwent instrument removal using the BTR-Pen system had less fracture resistance.

## 1. Introduction

The fracture of endodontic instruments presents a dilemma that every clinician deals with during root canal treatment [1]. The prevalence of instrument fracture has been reported between 1% and 5.1% [2,3,4,5]. Significantly more instrument fracture was observed in molars compared with premolars and incisors or canines. Moreover, it has been found to occur most frequently in the mesiobuccal roots of molars, due to the curvature and anatomy of the canal [2,3]

The probability of instrument fracture has been shown to be almost seven times higher for rotary instruments than for hand files [2], often with no evidence of plastic deformation [6]. NiTi rotary file fractures are caused by excessive cyclic flexural fatigue, torsional fatigue, or a combination of both. More recent evidence highlights that both characteristics interact with each other, and considered as a union, they are measured with the polar moment of inertia [7,8]; stainless steel instrument fracture is caused by excessive torque [9]. There are three orthograde treatment choices for the management of fractured instruments. The first two strategies maintain the fragment within the root canal, either by obturation up to the accessible part of the canal or by bypassing the fragment. The third treatment option is the retrieval of the broken instrument from the root canal [10]. Although removal of the broken instrument is the most favorable management option in cases with periapical involvement, a complete bypass might also provide a good prognosis by allowing the thorough debridement of the entire working length of the root canal in cases where retrieval is impossible [11]. The anatomy of the canal and the location of the broken instrument can make instrument removal stressful and time-consuming. Moreover, removal techniques may result in excessive loss of dentine, decreased root fracture resistance, and other complications, such as root perforation, extrusion of the fragment beyond the root apex, and an increase in the temperature of the external root surface [12,13,14]. The successful removal of broken instruments does not guarantee the success of the root canal treatment. Therefore, a clinician should find a balance between the successful removal of the broken instrument and the maintenance of the maximum amount of tooth tissue in terms of quality and quantity.

Various devices and techniques have been evaluated for the removal of separated instruments from the canal system [15]. The efficiency of ultrasonic devices and tips combined with a dental operating microscope (DOM) for the removal of dentine surrounding the fractured instrument and retrieval of the fractured fragments has been reported [15,16]. The Broken Tool Remover (BTR)-Pen system (Cerkamed Medical Company, Poland) is a recently introduced removal system [17]. The use of an ultrathin and highly elastic working tip, with an endurance nitinol loop, enables the possible grabbing of the broken instrument. It does not need excessive enlargement of the canal because of the shape memory, which allows to place it into the narrow and curved root canals. Our knowledge of the BTR-Pen system is largely based on very limited data, mostly from clinical cases, and there has been no research on the efficiency of the BTR-Pen in the literature.

The limitations of current techniques have led to the search for more efficient broken instrument removal techniques that may optimize the efficiency of the retrieval procedure and eliminate the negative effects on the tooth structure and strength of the root. Therefore, the aims of this study were to evaluate the efficiency of using the BTR-Pen system in removing different types of instrument fragments from root canals and assess its effect on the fracture resistance of the root after the removal of the instrument and compare it with the use of ultrasonic tips alone.

## 2. Materials and Methods

This study was carried out at the Kocaeli University Faculty of Dentistry Department of Endodontics, Kocaeli, Turkey by obtaining the approval of the Non-Invasive Clinical Research Ethics Committee of Kocaeli University Faculty of Medicine (KÜ GOKAEK 2020315).

### 2.1. Selection and Preparation of Samples

In this study, teeth were selected from a pool of recently extracted teeth, and 130 maxillary molars having inclusion criteria were included in the study. The roots were checked radiographically using preoperative periapical films and visually under the DOM (OPMI PICO; Carl Zeiss, Göttingen, Germany) for inclusion criteria. The teeth with former root canal therapy were excluded. The selected teeth had closed apices, no root caries, anomalies, fractures, or cracks, and no signs of internal or external resorption. The curvature angles of mesiobuccal roots on the mesio-distal and bucco-palatal views were measured using a computer program (Turcasoft Software and Industry Limited Company, Samsun, Turkey 2012). The curvature angle for each mesiobuccal root was verified to be ≤20° according to Schneider’s [18] method. The roots having greater curvature angles were excluded. The roots were cleaned ultrasonically, and the teeth were kept in a 10% formalin solution.

The crowns were removed, and the roots were separated using a diamond disc, leaving 11 mm long mesiobuccal roots. The mesiobuccal roots (*n* = 130) were used throughout the study. Access cavities were prepared. Patency was established in the canals using #10 K-files.

An endodontic resident instrumented all specimens using K-files (VDW, Munich, Germany) and NiTi rotary instruments (ProTaper, Dentsply, Maillefer, Ballaigues, Switzerland), using a crown-down approach up to size #25 and F2 (#25, 0.06 taper), respectively. The root canals were irrigated with 2 mL of 2.5% NaOCl between instruments.

Ten roots (*n* = 5 per group) were allocated to two control groups for the fracture resistance test. Instrument separation and removal procedures were not applied to the roots in the control groups. These groups were used to establish baseline values for the root-fracture resistance test. In the first control group, no procedure was performed after instrumentation. In the second control group, root canal obturation was performed as follows: after shaping the samples, the root canals were dried using paper points (Dentsply Maillefer, Ballaigues, Switzerland) and were obturated with gutta-percha (Diadent, Choongchong Buk Do, Korea) and AH Plus canal sealer (Dentsply-DeTrey, Konstanz, Germany) using the lateral compaction technique. The canal orifices of all samples were sealed with a temporary filling material (Detax, Ettingen, Germany). The roots in the control groups were stored at 37 °C and 100% humidity until the fracture resistance test.

A total of 120 roots were divided into four subgroups (*n* = 30 each) according to the types of instruments fractured (K-files and NiTi rotary instruments) and the location of the fractured instrument (middle and apical third). K-file size #25 or ProTaper F2 instruments were notched to a depth of half the instrument thickness at a point 3 mm from the tip using a diamond bur. The endomotor was rotated clockwise with pressure until the instruments separated in either the apical or middle thirds of the roots. All samples were stored at 37 °C and 100% humidity.

### 2.2. Broken Instrument Removal Procedures

A ‘staging platform’ was created to increase the visibility of the broken instrument. Gates Glidden drills were modified by cutting the guiding tips with a diamond bur at their maximum cross-sectional diameters to permit the preparation of a circumferential staging platform. Gates Glidden drills #1, #2, and #3 were used sequentially until they made contact with the most coronal part of the separated instrument. An RT2 ultrasonic tip (EMS, Sybron Endo, Switzerland) was used to trephine the dentine around the coronal tip of the broken instrument.

**Scheme 1:**
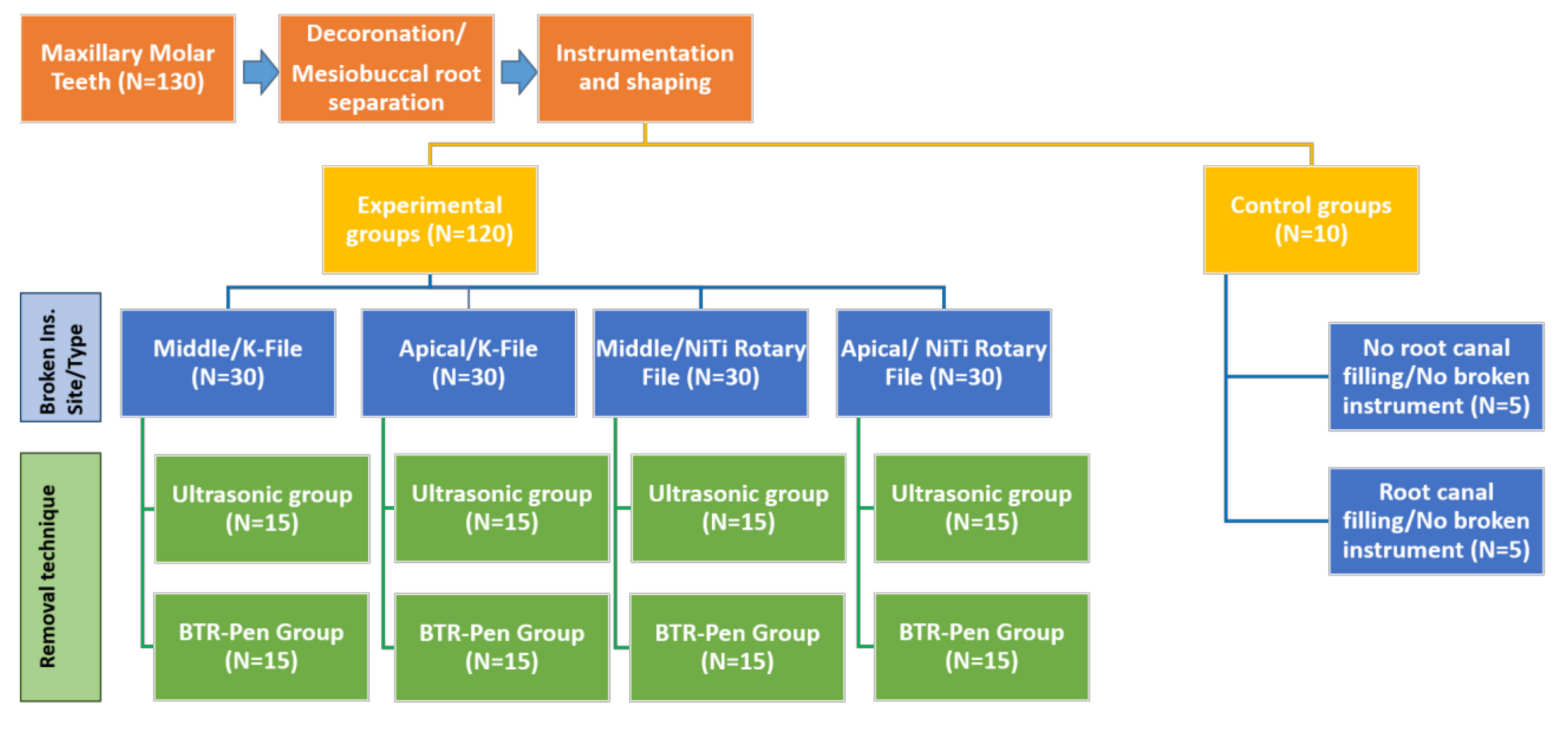
Classification of groups.

For the removal process, the specimens of the four main groups were further subdivided into two subgroups (*n* = 15 each) according to the technique used (Scheme 1).

**Figure 1:**
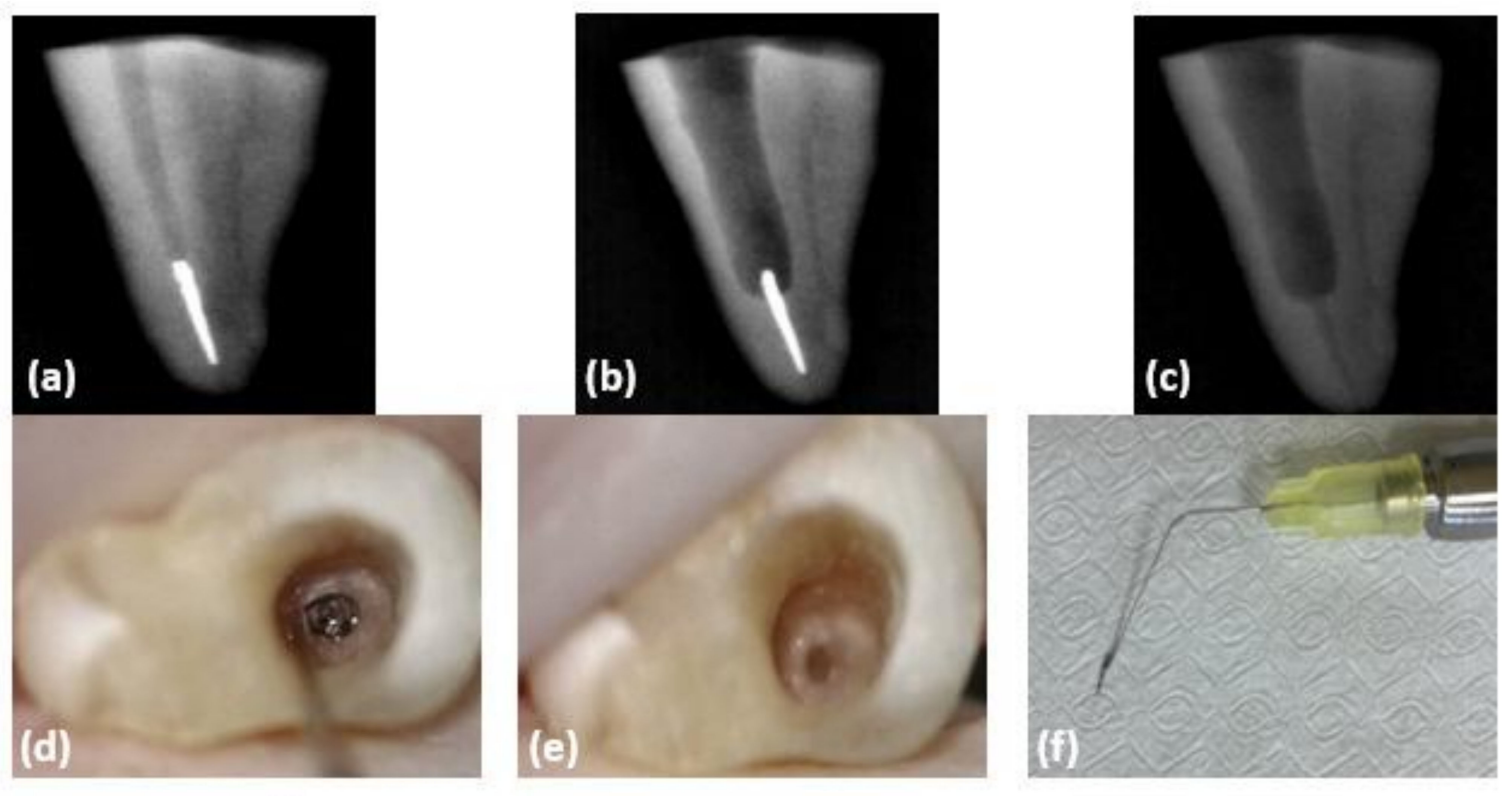
(**a**) Fractured fragment located in the apical third. (**b**) Trephining of dentine around the coronal 1.5 mm of the broken instrument. (**c**) After the removal of the fractured fragment. (**d**) Before and (**e**) after removal of the fractured fragment using the BTR-Pen under the dental operating microscope at 10×. (**f**) The broken fragment was gripped by the BTR-Pen loop.

The separated instruments were removed from the root canal using the procedures described below:

(1) Ultrasonics technique: An ultrasonic device (EMS miniMaster Piezon, Nyon, Switzerland) and RT3 ultrasonic tips (EMS, Sybron Endo, Switzerland) were used to remove the broken instruments. The RT3 tip was used without water cooling and rotated counterclockwise around the broken instrument.
(2) The BTR-Pen system: Working tips with a 0.3 mm diameter loop were used. The tip with a nitinol loop was bent according to the bending angle of the root canal. The loop was placed over the broken file, and the coronal 1.5 mm of the broken instrument was grasped with the BTR-Pen loop (Cerkamed Medical Company, Stalowa Wola, Poland) (LOT No: 1911201), squeezed by moving the slider’s cap up, and the instrument was extracted from the root canal (Figure 1).

The operator was trained to use the BTR-Pen system before the experiment. All procedures for the removal of instruments were performed under a DOM. Successful removal was determined as the complete removal of the fractured instrument from the root canal without creating a root perforation. A 45 min time limit was set for the removal process.

In the experimental groups, a 25 # K-file was used to verify the patency of the root canal after the removal of the broken instrument and to ensure that no fragment was left within the canal. The removal time was recorded from the start of staging platform preparation.

### 2.3. Preparation for Mechanical Testing

The apical 3–4 mm of the root covered with a 0.2 mm layer of polyether impression material (Impregum Garant L DuoSoft, 3M ESPE, Seefeld, Germany) was vertically embedded in an acrylic resin block with respect to the horizontal plane. The acrylic blocks with the mounted roots were left for 24 h for the complete setting of the resin. A custom-made steel jig (1.5 mm diameter tip) was attached to the loading cell of a universal testing machine (Instron 4411, High Wycombe, UK). The acrylic resin block with the embedded root was fixed in the testing machine in such a way that the steel jig traveled axially at a 90° angle toward the center of the root filling at a crosshead speed of 1 mm/min until the root fractured. The fracture was defined as the point at which a sharp drop in the force was observed. The maximum breaking loads were recorded in Newtons by a computer connected to the testing machine.

### 2.4. Statistical Analysis

The success rate of the removal of the fractured instruments, operating time, and fracture resistance of the root were analyzed statistically using the MedCalc Statistical Software Version 12.7.7 (Ostend, Belgium). Continuous variables belonging to more than two groups that did not show normal distribution were compared using the Kruskal–Wallis test. Post-hoc pairwise comparisons of variables found to be significant in multiple comparisons were performed using the Mann–Whitney U test. The relationship between two continuous variables not suitable for normal distribution was evaluated using Spearman’s rho correlation test. The level of significance was set at *p* < 0.05.

## 3. Results

**Figure 2:**
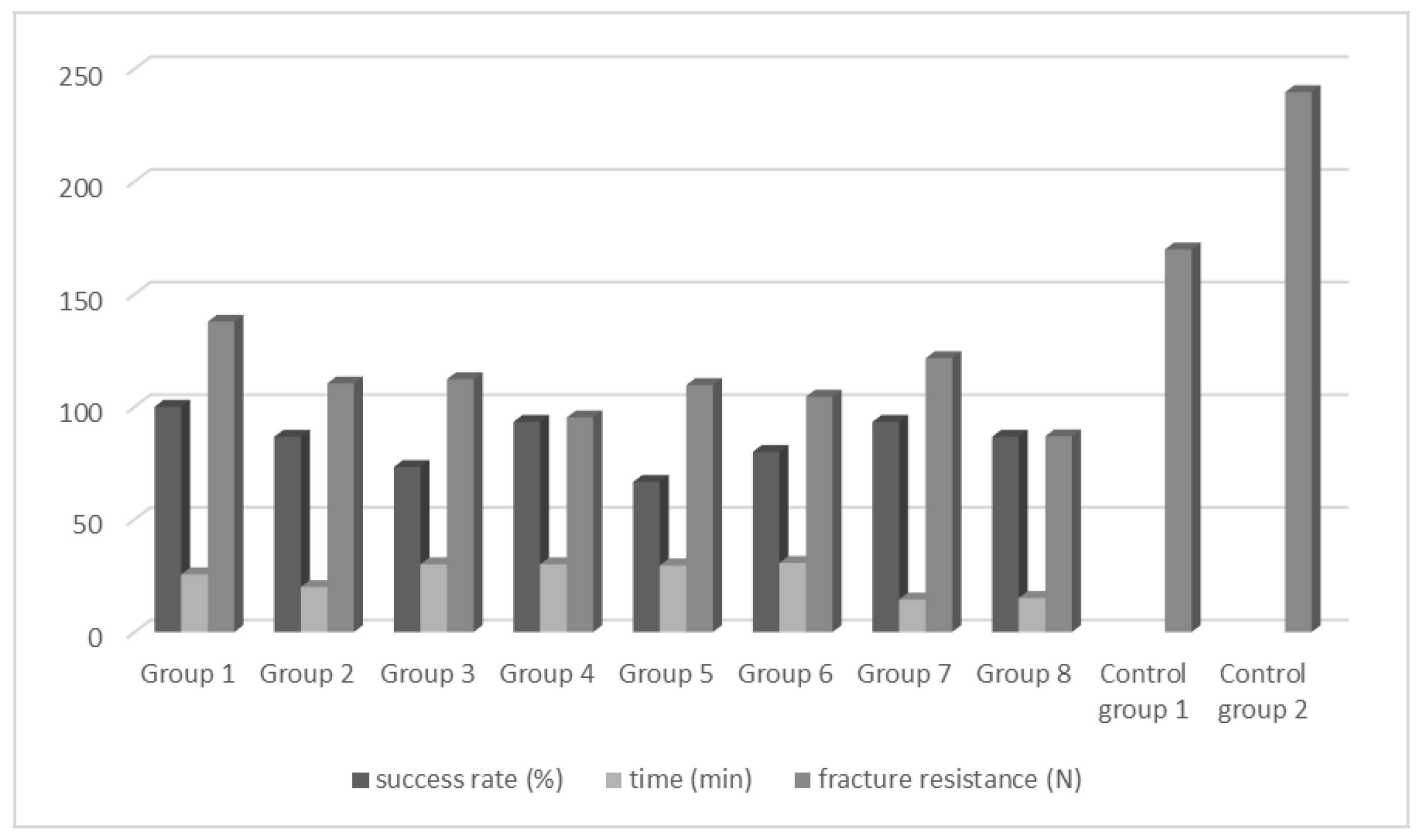
Comparison of success rate (%), removal time (min), and force required for root fracture (N) between the experimental and control groups.

The overall success rate achieved in removing broken instruments from root canals was 85% (102/120). When evaluating the overall success of each removal technique, the BTR-Pen system successfully extracted 52 of 60 broken instruments (success rate: 86.7%), whereas the ultrasonic technique resulted in the successful extraction of 50 out of 60 broken instruments (success rate: 83.3%). However, there were no statistically significant differences between the two techniques (*p* > 0.05). There was no statistically significant difference between all eight subgroups in terms of success rate (*p* > 0.05) (Figure 2). In the apical region, the BTR-Pen system had a higher success rate (86.7%) than the ultrasonic technique (70%). However, this difference was not statistically significant (*p* > 0.05).

The overall success rate of instrument removal from the apical third was 78.3%, and for the middle third, it was 91.7% (*p* > 0.05). With regard to fractured K-files, 88.3% (53/60) were successfully removed, whereas in cases of broken NiTi instruments, the success rate was 81.7% (49/60). The difference between success rates according to instrument type was not statistically significant (*p* > 0.05).

The average time required for removing file fragments was 24.03 ± 8.3 min. When the time needed for removing separated fragments using each removal technique was compared, the BTR-Pen system (23.97 ± 8.35 min) showed similar results to that of the ultrasonic technique (24.1 ± 8.28 min; *p* > 0.05). There was a significant statistical difference between the time needed to remove separated fragments from the middle (18.82 ± 6.3 min) and apical thirds (30.13 ± 5.8 min) (*p* < 0.05). Instrument retrieval using the BTR-Pen was faster (17.57 ± 3.85 min) in the middle third of the root canal. However, the time difference between the BTR-Pen system and ultrasonic technique was not statistically significant (*p* > 0.05). The time needed for the removal of fractured K-files (26.18 ± 7.2 min) was significantly higher than that required for the removal of fractured NiTi (21.7 ± 8.8 min) instruments.

When all eight subgroups (Scheme 1) were compared, there was a statistically significant difference between them in terms of removal time (one-way analysis of variance, *p* < 0.05). The average time in group 1 was higher than those in groups 7 and 8 (Tukey’s *p* < 0.001, Bonferroni correction). The average time in group 2 was lower than those in groups 3, 4, 5, and 6 (Tukey’s *p* < 0.001, Bonferroni correction). The average times in groups 4, 5, and 6 were higher than those in groups 7 and 8 (Tukey’s *p* < 0.001, Bonferroni correction).

The forces required for vertical fractures in the groups are presented in Table 1. The highest mean force was recorded in control group 2 (239.7 ± 147.5 N), followed by control group 1 (169.9 ± 72.1 N). The force required in control group 2 was significantly higher than that in the experimental groups (*p* < 0.05). There was a significant difference in the force required for root fracture among the BTR-Pen and ultrasonic groups (*p* = 0.024), with roots in the ultrasonic group having greater fracture resistance.

The mean force required for root fracture did not significantly differ between roots with file separation in the apical and middle thirds (104.4 ± 35.5 N and 115.2 ± 65.9 N, respectively; *p* > 0.05). The removal of K-files (114.6 ± 64.1 N) or NiTi (105.6 ± 40.8 N) instruments did not significantly affect the fracture resistance of the teeth (*p* > 0.05). When all eight subgroups were considered, the fracture resistance of control group 2 was significantly higher than that of groups 4 and 8 (Tukey’s *p* < 0.001, Bonferroni correction).

## 4. Discussion

Instrument separation during root canal treatment prevents the effective cleaning and shaping of the root canal [19]. This may reduce the success rate of endodontic treatment. Therefore, the removal of broken instruments without complications, followed by the cleaning and shaping of the root canal system, is the most appropriate approach [20]. Various methods have been developed to remove broken instruments from the root canal. Ruddle [21] reported the preparation of a staging platform and trephining the coronal dentine around broken fragments using ultrasonic tips. The ultrasonic technique combined with a DOM increases the success rate and safety of the removal of fractured instruments [4]. The approach to removing fractured instruments from the canal should always include techniques and devices that remove minimum dentine, resulting in a higher success rate in a shorter time [22]. The BTR-Pen system was introduced recently and has been designed to be placed in narrow and curved canals. To the best of our knowledge, the present study is the first to evaluate the efficiency of the BTR-Pen system in the removal of broken instruments. The retrieval of different types of broken instruments from different regions of roots was evaluated and compared between the BTR-Pen system and ultrasonic tips alone.

The removal of fractured instruments is a difficult and time-consuming procedure, and the success rate can vary considerably. Previous studies reported removal success rates of ultrasonics between 80% and 88% [23,24,25]. These rates correlate well with our findings. The technique used during the process may be a key factor [11,26]. To date, none of these techniques have achieved absolute success. Shen et al. [26] reported an overall success rate of 53% in the removal of NiTi instruments using a variety of techniques and armamentarium. Hülsmann and Schinkel [1] reported a success rate of 68% in a retrospective study in which they included bypassed instruments. In contrast, another study demonstrated a success rate of 76.47% using a trephine bur/microtube technique [20]. Suter et al. [23] achieved an overall success rate of 87% in removing different types of broken instruments using ultrasonics and the tube and Hedström file method. There are a variety of acceptable treatment options for achieving success in clinical conditions. The reason for these contradictory results in the literature might be the different study designs and definitions of success in clinical studies and the lack of clinical conditions in in vitro experiments.

The successful removal of broken instruments depends on the material type, location, and length of the instrument [26]. When the successful removal of broken instruments was evaluated according to location, the success rates in the apical and middle thirds were 78.3% and 91.7%, respectively. In clinical studies, Suter et al. [23] reported success rates of 87.50% and 87.10%, while Cujé et al. [25] reported success rates of 93% and 100% in the apical and middle thirds, respectively. These variations in success rates can be attributed to time limitations and the use of different samples and instruments.

Because the instrument type is an important factor affecting instrument removal, we included both NiTi rotary instruments and K-files in the study design. Shen et al. [26] reported success rates of 53% and 59% for removing broken NiTi rotary instruments and stainless steel files, respectively. On the other hand, Cujé et al. [25] achieved success with 89% of fractured rotary instruments and 100% of fractured stainless steel files. In the present study, 88.3% of the K-files and 81.7% of the NiTi rotary instruments were successfully removed. This inconsistency may be attributed to the experimental group consisting of 120 samples, which may be due to the K-type file being the only stainless steel instrument used in the study. However, similar to other studies [23,26], this study revealed that the type of instrument did not affect removal success.

If the broken instrument is short, it is difficult to remove it from the canal [1]. In accordance with previous studies [10,27,28], the length of the broken instrument was chosen as 3 mm in this study. However, the impact of fragment length on the retrieval process was beyond the scope of this study. Nonetheless, the technique of the BTR-Pen system suggests that it would be easier to extract a longer fragment. Further data on grabbing fragments of different lengths with the BTR-Pen are needed to determine its exact efficiency.

In the present study, the crowns were removed, and only the mesiobuccal canals of the maxillary molar teeth were used. Tooth fracture resistance has been an established method of investigation of the effect of cavity/restoration design on tooth strength. The force applied to the tooth is affected by factors involving both the root and crown [10]. Various factors such as access cavities [27], restorative materials, post-placement, and prosthetic crowns can affect the fracture strength of teeth. The larger bite forces may be more likely to cause tooth fracture. [29] Shahabinejad et al. [10] removed the crowns before experimentation to avoid the effect of coronal factors in their study.

With regard to the removal time, Meng et al. [20] reported that an average of 8.55 ± 5.81 min was required for the successful removal of broken instruments using the trephine bur/microtube technique. Madarati et al. [30] reported that 14.8 min and 21.2 min, and 14.4 min, were the average times required for removing instruments using ultrasonics from the middle and apical thirds, respectively. The time required for removing instruments was 18.82 ± 6.3 min and 30.13 ± 5.8 min in the middle and apical thirds, respectively, and the average instrument removal time using ultrasonic and BTR-Pen techniques were 24.03 ± 8.3 min. However, the sample type, number, and extraction method may have affected the working time.

Excessive loss of tooth substance can reduce root fracture resistance in endodontically treated teeth [12,14]. This might lead to the extraction of single-rooted teeth and the amputation or hemisection of multi-rooted teeth and periodontal disease [31,32]. The process of gaining access to the fractured instrument and removing it involves dentine removal, regardless of the technique used. In line with this, all experimental groups had lower fracture resistance compared to the control groups in our study. Therefore, attention must be paid to the potential dentine loss during broken instrument removal. The ultrasonic group demonstrated greater fracture resistance than the BTR-Pen group. This finding might be attributed to the need of additional ultrasonic trephining to ease the placement of BTR-Pen loops around the instrument fragment in some samples, depending on the complexity. Moreover, this finding represents merely the load capacity of the investigated samples. Fracture resistance studies based on static loading more likely represents the critical stress amplitude for one cycle; it is unlikely to make long-term failure predictions. [33] We believe that our research will serve as a base for future studies, which may take all intraoral (biological, physical, and chemical) challenges into account. Souter and Messer [14] showed that instrument removal from the middle (30%) and apical (40%) thirds significantly weakened the root compared to the control group. Gerek et al. [27] showed that the removal of fractured instruments from the middle third of the anterior teeth decreased root strength by 30%–40% relative to that in the control group. These values correlate well with the findings of the present study, that the fracture resistance of teeth with a broken instrument extracted from the middle third decreased by 32% compared to the first control group.

In the present study, some difficulties and complications were encountered while using the BTR-Pen system. It was observed that when the ultrasonic tip contacted the broken instrument, it wore it down and shortened its length. A similar finding was reported by Terauchi et al. [22] and Hülsmann and Schinkel [1]. Because of the abrasion of the broken instrument, more dentine had to be removed with an ultrasonic tip before the placement of the BTR-Pen loop around the fractured fragment. In addition, if the fragment was too tight in the canal, it was loosened further by ultrasonic tips until a dance movement was observed. Increased dentine loss is not particularly surprising if we consider that only one technique was consistently used in each root. Therefore, we suggest that various techniques and management strategies should be chosen or combined according to the complexity of the clinical situation.

The present experimental study was designed to standardize the test conditions and control confounding test variables. However, there are limitations with the experimental study design, which cannot replicate the real clinical scenario [34]. The samples were prepared free of the coronal tooth structure in order to eliminate the coronal factors affecting the strength of the tooth. This design allowed us to focus on the factors influencing only the root structure. However, it allowed unrestricted access to the cavity and direct vision of the fragment. The lower success rate in clinical situations could be attributed to coronal interference and restricted visualization. Another limitation of this study was the mode of separation and location of the separated instrument fragment. Instrument fractures occur mostly in the apical third of the root canal [10]. To separate the instruments intentionally in different locations, the Protaper F2 instruments were notched at the predetermined points and rotated in the canal with pressure. When the apical third was considered, failure following the stresses and strain imposed on the instrument seemed more likely to correlate with a clinical situation. However, the friction of the fractured tip may not replicate the friction of an inadvertently fractured instrument in the middle third, which may lead to failure exclusively because of flexural stresses. On the other hand, in clinical practice, both torsional and flexural stresses occur simultaneously, and flexural failure is especially observed in curved root canals. In this regard, the experimental model of the present study differs from the complexity of the separation process in a clinical scenario where all the factors and variables act simultaneously, leading to metal fatigue and failure [35]. Moreover, a reduced mouth opening might pose further problems in a clinical situation and accordingly influence the success rate and the management time. Despite this, we believe that our data could be a starting point for further studies to assess the efficiency of the BTR-Pen, and more clinical studies have yet to be carried out.

The treatment prognosis is affected by many factors, such as the root canal shaping stage, the level of microbial contamination, and the location of the fractured instrument [36]. Decisions about broken fragment management and removal choices should be carefully assessed, considering all these factors. Terauchi et al. [22] suggested that in cases where the broken instrument cannot be removed, the broken fragment can be included in the canal filling. Madarati et al. [37] stated that leaving fractured instruments in the apical third of the canal would not affect root resistance to vertical fractures. Therefore, the treatment option should be selected after evaluating its benefits and the risks due to the loss of dentine.

## 5. Conclusions

The results of this study suggest that neither of the broken instrument removal techniques tested was superior to the other in terms of overall success rate and removal time. The location and type of broken instrument did not significantly affect the success rate of retrieval but appeared to affect the removal time. The roots in the ultrasonic group required greater force for root fracture than those in the BTR-Pen group. Further research should focus on identifying a quick and effective retrieval procedure that minimizes dentine damage.

## Figures and Tables

**Table 1:** Comparison of the fracture resistance of the experimental groups, using two different techniques with the control groups.

	1st Control	2nd Control	BTR-Pen	Ultrasonic
	Mean ± SD Med. (Min.–Max.)	Mean ± SD Med. (Min.–Max.)	Mean ± SD Med. (Min.–Max.)	Mean ± SD Med. (Min.–Max.)
Fracture Resistance	169.9 ± 72.1 195.3 (88.5–254.2)	239.7 ± 147.5) 257.5 (80.4–420.5)	98.9 ± 45.7 * 91.4 (40.9–336.1)	122.5 ± 60.1 107.3 (17.4–374.5)

Mann–Whitney U test, * *p* < 0.05.

## Data Availability

The data in this study are available upon request from the corresponding author.
